# Fluorescence microscopy as an alternative to electron microscopy for microscale dispersion evaluation of organic–inorganic composites

**DOI:** 10.1038/ncomms11811

**Published:** 2016-06-02

**Authors:** Weijiang Guan, Si Wang, Chao Lu, Ben Zhong Tang

**Affiliations:** 1 State Key Laboratory of Chemical Resource Engineering, Beijing University of Chemical Technology, 15 Beisanhuan East Road, PO Box 98, Beijing 100029, China; 2 Department of Chemistry, Hong Kong Branch of Chinese National Engineering Research Center for Tissue Restoration and Reconstruction, Hong Kong University of Science and Technology, Clear Water Bay, Hong Kong 999077, China

## Abstract

Inorganic dispersion is of great importance for actual implementation of advanced properties of organic–inorganic composites. Currently, electron microscopy is the most conventional approach for observing dispersion of inorganic fillers from ultrathin sections of organic–inorganic composites at the nanoscale by professional technicians. However, direct visualization of macrodispersion of inorganic fillers in organic–inorganic composites using high-contrast fluorescent imaging method is hampered. Here we design and synthesize a unique fluorescent surfactant, which combines the properties of the aggregation-induced emission (AIE) and amphiphilicity, to image macrodispersion of montmorillonite and layered double hydroxide fillers in polymer matrix. The proposed fluorescence imaging provides a number of important advantages over electron microscope imaging, and opens a new avenue in the development of direct three-dimensional observation of inorganic filler macrodispersion in organic–inorganic composites.

## Abstract (web summary)

Imaging of inorganic dispersions in organic-inorganic composites is typically performed using electron microscopy. Here, the authors show that surfactants with attached aggregation-induced emission fluorophores allow simple fluorescence imaging of the spatial distribution of the inorganic filler dispersion.

## Graphical abstract

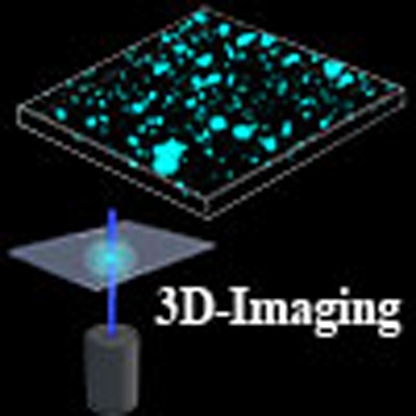

Since the birth of organic–inorganic composites, they have undoubtedly been one of the most important and active scientific fields^1^,^2^,^3^,^4^,^5^. For such composite materials, the dispersion state of inorganic fillers in organic matrix plays a vital role for attainable improvements of their properties^6^,^7^,^8^,^9^. The dispersion–property relationship can be understood through the direct observation of the spatial distribution of inorganic fillers. The conventional observation method for inorganic filler microdispersion in organic matrix is performed by electron microscopy techniques, such as transmission electron microscopy (TEM) and three-dimensional (3D)-TEM tomography^6^,^7^,^8^,^9^,^10^,^11^,^12^,^13^,^14^. Notwithstanding the impressive amount of their data on microdispersion of inorganic fillers, they suffer from their own intrinsic limitations. First, the sample preparation is very complex and time-consuming, requiring professional technicians to cut and thin with special care; second, it is only suitable for evaluating nanodispersion scale in a small area window, and thus the obtained results could not be truly representative of macrodispersion of inorganic fillers; in addition, heavy-element staining is required for some poor-contrast components^10^,^11^,^12^,^13^,^14^. Therefore, it appears as a crucial need to develop a preparation-free, operation-simple and high-contrast method for visualization of macrodispersion of inorganic fillers.

The ultrafast and non-invasive 3D visualization is one of native functionalities for confocal fluorescence microscopy (CFM)^15^,^16^,^17^. In principle, CFM would be a powerful method for analyses of inorganic filler macrodispersion and spatial distribution in organic matrix with a large-enough window if the inorganic fillers could emit light. However, the fluorescence quenching usually occurs upon the formation of the fluorophore aggregates in composite materials via *π*–*π* stacking interactions^18^,^19^. Therefore, the direct visualization of macrodispersion of inorganic fillers in organic–inorganic composites using high-contrast 3D fluorescent imaging method is hampered. Such a limitation of fluorescent labelling should be overcome by means of the aggregation-induced emission (AIE)-active fluorophores, which are generally non-fluorescent in solution but induced emission highly in the aggregated state or solid state^20^,^21^,^22^,^23^,^24^.

Herein, we choose a typical organic–inorganic composite, montmorillonite (MMT) polymer composite, to achieve this possibility. In general, the naturally occurring MMT is hydrophilic, and requires organic modification by intercalating cationic surfactants into the interlayer space through ion exchange to form organically compatible^25^,^26^,^27^,^28^. The organo-modified MMT can be well dispersed in polymer matrix with remarkable improvement of material properties, such as increased strength and heat resistance, decreased gas permeability and flammability, and increased biodegradability^29^,^30^,^31^,^32^,^33^. It is anticipated that organo-modified MMT can emit light if the intercalated cationic surfactants are attached with an AIE-active fluorophore. In this work, we first synthesize tetraphenylethene (TPE)-cored dodecyltrimethylammonium bromide cationic surfactant (denoted as TPE-DTAB) by incorporating a typical AIE-active TPE luminophore^34^,^35^,^36^ into DTAB. Furthermore, we demonstrate the feasibility of TPE-DTAB as a novel probe for visualization of MMT macrodispersion and spatial distribution in polyvinyl chloride (PVC) matrix with some unique advantages, such as preparation-free, ultrafast and non-invasive (Fig. 1). The generality of the present strategy has also been verified by direct visualization of macrodispersion of layered double hydroxide (LDH) fillers in PVC matrix. This work also serves to demonstrate the general potential in the use of suitable AIE-active luminophores for the visualization of the filler macrodispersion in other organic–inorganic composites.

## Results

### Characterizations of TPE-DTAB

**Figure 2:**
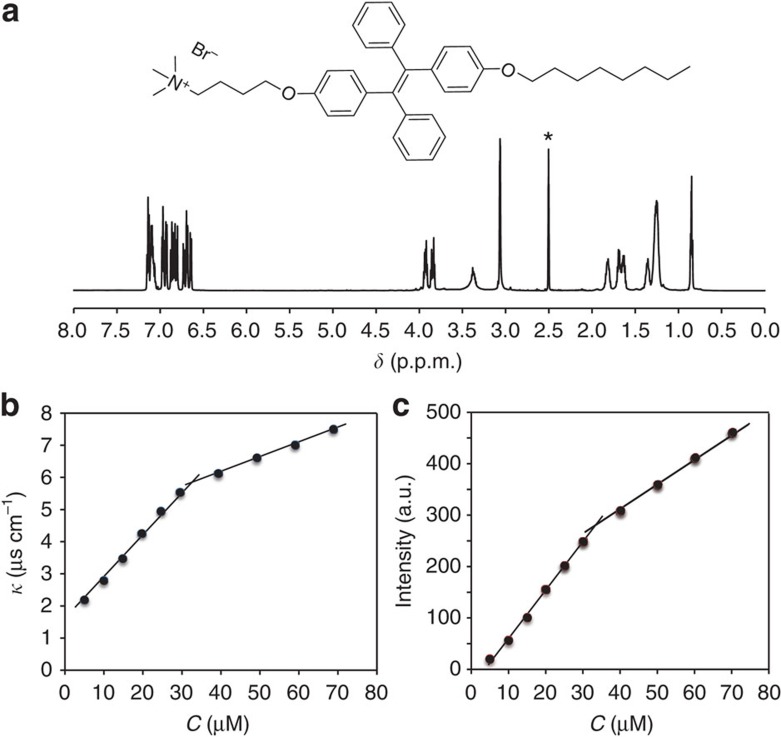
Molecular structure and characterization of TPE-DTAB. (**a**) ^1^H NMR spectrum of TPE-DTAB in [D_6_]dimethyl sulfoxide (the solvent peak is marked with asterisk). Plots of conductivity (**b**) and fluorescence intensity at 490 nm (**c**) versus the concentration of TPE-DTAB. *λ*_ex_=325 nm.

As depicted in Supplementary Fig. 1, in our synthetic strategy for TPE-DTAB surfactant, we first prepared a fluorescent TPE core with two hydroxyl groups (**1**, TPE-2OH) to allow access to alkyl chain and alkyl bromide, respectively^37^. Then TPE-2OH was treated with equivalent moles of NaH to activate a hydroxyl group of TPE-2OH, followed by introducing n-octane in one side of TPE-2OH to yield compound **2** (ref. 38). After removal of the unreacted TPE-2OH, the reaction between 1,4-dibromobutane and compound **2** was performed in the presence of alkaline K_2_CO_3_ to generate bromo-functionalized compound **3** (ref. 39). The molecular structures of the above intermediate compounds were characterized and verified by ^1^H nuclear magnetic resonance (NMR) spectroscopy (Supplementary Figs 2–4). Next, the alkyl bromide of compound **3** was converted to the alkyl trimethylamine bromide by adding excess amount of trimethylamine. Finally, TPE-DTAB (**4**) was successfully synthesized^40^. From the ^1^H NMR spectrum of the purified TPE-DTAB (Fig. 2a), the characteristic peaks of TPE were clearly visible, and the other peaks corresponded nicely to the alkyl protons of DTAB. In addition, in the positive-ion mode mass spectrum (MS; Supplementary Fig. 5), a mass-to-charge (*m*/*z*) ratio of 590.3996 was found to be consistent with the exact mass of TPE-DTAB without bromide ion. Finally, in combination with the measurement of the ^13^C NMR spectrum (Supplementary Fig. 6), we confirmed the structure of the cationic TPE-DTAB.

The critical micelle concentration (CMC) value of the cationic TPE-DTAB surfactant solution was determined by electrical conductivity method^41^,^42^,^43^. The conductivities (*κ*) of the TPE-DTAB at different concentrations (*C*) were plotted in Fig. 2b. At low concentrations, the solution conductivity increased linearly with an increase in the concentration of TPE-DTAB up to 32 μM. However, when the TPE-DTAB concentrations were above 32 μM, the straight line was observed with a decreased slope. The change of the slope was ascribed to the fact that the ionic micelles have less charge per unit mass than their unimers^41^,^42^,^43^. The breakpoint at ∼32 μM is generally considered to be the CMC of the TPE-DTAB surfactant.

The optical properties of the TPE-DTAB surfactant solution were investigated by measuring its ultraviolet−visible absorption and fluorescence spectra. As shown in Supplementary Fig. 7, two absorption peaks appeared at about 251 and 320 nm, which were attributed to the absorption of benzene and TPE unit^44^. On the other hand, the fluorescence emission wavelength remained at 490 nm, indicative of a typical TPE pattern of light^34^,^35^,^36^,^44^. The fluorescence intensity of the TPE-DTAB surfactant solution at 490 nm was plotted as a function of the TPE-DTAB concentration (Fig. 2c). Two straight lines with different slopes in the intensity–concentration plot were found with an inflection point at ∼32 μM, indicating the changes of the TPE-DTAB morphology^45^. Moreover, ultraviolet absorption and photoluminescence excitation spectra of TPE-DTAB were recorded below and above CMC (Supplementary Fig. 8). It can be seen that absorbance is simply proportional to photoluminescence species. However, inner filter effect could happen if absorbance is higher than 0.05, causing disproportional increasing of photoluminescence intensity with the increase of the concentration of photoluminescence species^46^. Experimentally, we obtained the linear relationship between TPE-DTAB concentration and photoluminescence intensity. The result may be ascribed to the combined effects, including inner filter effect, AIE effect and some other uncertain effects. The resulting CMC value by fluorescence measurement matched well with that obtained from the conductivity measurement, demonstrating that the synthesized TPE-DTAB is a kind of cationic fluorescent surfactant.

### Characterizations of TPE-DTAB-modified MMT

**Figure 3:**
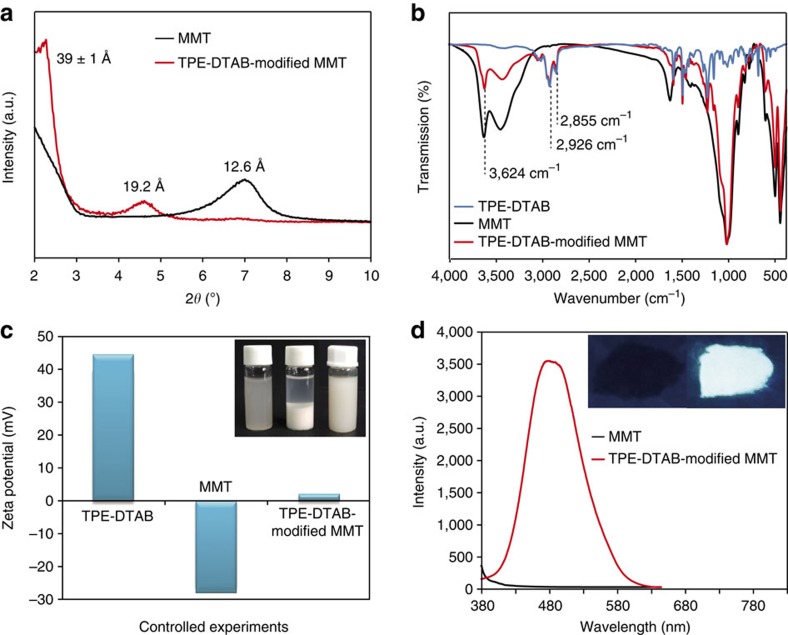
Characterizations of TPE-DTAB-modified MMT. (**a**) Powder X-ray diffraction patterns of Na^+^-MMT and TPE-DTAB-modified MMT. (**b**) Fourier transform infrared spectra of TPE-DTAB, Na^+^-MMT and TPE-DTAB-modified MMT. (**c**) *ζ* potential measurements of TPE-DTAB, Na^+^-MMT and TPE-DTAB-modified MMT; the inset showed the photographs of Na^+^-MMT in water, TPE-DTAB-modified MMT in water and TPE-DTAB-modified MMT in petroleum ether for 24 h, respectively. (**d**) Fluorescence spectra of Na^+^-MMT and TPE-DTAB-modified MMT; the inset showed the photographs of Na^+^-MMT powder (left) and TPE-DTAB-modified MMT powder (right) under ultraviolet irradiation at 365 nm.

It is known that Na^+^-MMT is hydrophilic and well dispersed in polar solvent like water; however, the agglomeration of Na^+^-MMT particles is inevitably occurred when they are mixed with organic polymer matrix to fabricate organic–inorganic composites. To obtain hydrophobic MMT, the TPE-DTAB was intercalated into the interlayer space of Na^+^-MMT via ion exchange method^25^,^26^,^27^,^28^. The X-ray diffraction patterns of Na^+^-MMT and TPE-DTAB-modified MMT were measured to study the structure change of MMT. It is known that 2*θ* change in the range of 2°–10° for layered silicates indicated the formation of new ordered intercalated cationic layers^47^,^48^,^49^. As shown in Fig. 3a, the (001) diffraction peak of Na^+^-MMT occurred at 7.00°, reflecting a *d*-spacing (d_001_) of 12.6 Å. Interestingly, the (001) peak of the TPE-DTAB-modified MMT was shifted to 4.61° and 2.27°, indicating an enlarged *d*-spacing of 19.2 and 39±1 Å, respectively, suggesting that the MMT interlayers might be intercalated with TPE-DTA^+^ ions in different arrangements (Supplementary Fig. 9)^47^,^48^,^49^.

Fourier transform infrared spectra of the TPE-DTAB, the Na^+^-MMT and the TPE-DTAB-modified MMT further confirmed the combination of the TPE-DTA^+^ ions and the MMT particles (Fig. 3b). In comparison with the Na^+^-MMT, the TPE-DTAB-modified MMT exhibited two new absorption bands at 2,926 and 2,855 cm^−1^, which were ascribed to the asymmetrical and symmetrical stretching vibrations of methylene groups in the alkyl chains of TPE-DTAB, respectively^50^. Moreover, the absorption intensity at 3,624 cm^−1^ of the TPE-DTAB-modified MMT was lower than that of the Na^+^-MMT because of the improved hydrophobicity^51^. In conclusions, the TPE-DTA^+^ ions can be inserted into the interlayers of MMT.

On the other hand, the inorganic cations at the external surfaces of MMT particles would be also replaced by TPE-DTA^+^ ions as a result of the ion exchange reaction between TPE-DTAB and Na^+^-MMT. It is known that the adsorption of ionic surfactants at the particle surface usually has a great impact on the *ζ* potential of the particle^52^,^53^,^54^,^55^. Therefore, to clarify the configuration of TPE-DTAB at the MMT surfaces, we measured the *ζ* potential of the TPE-DTAB, Na^+^-MMT and TPE-DTAB-modified MMT, respectively (Fig. 3c). The TPE-DTAB showed a positive *ζ* potential of 43.5 mV, whereas the Na^+^-MMT particles had the negatively charged surfaces with a negative *ζ* potential of −28.8 mV. For the TPE-DTAB-modified MMT, its *ζ* potential was changed to approximately zero, indicating that TPE-DTA^+^ ions were adsorbed at the MMT surface via electrostatic attraction to expose their hydrophobic tails to the aqueous environment^55^. In addition, the Na^+^-MMT particles dispersed well in water; however, the TPE-DTAB-modified MMT particles seemed to be very swollen and hydrophobic. In comparison, the hydrophobic property of TPE-DTAB-modified MMT particles were further confirmed via their good dispersion in petroleum ether (PE) for 24 h (inset of Fig. 3c).

It is essential to study the fluorescence property of TPE-DTAB-modified MMT in the solid state. Figure 3d showed the fluorescence spectra of the Na^+^-MMT powder and the TPE-DTAB-modified MMT powder. The TPE-DTAB-modified MMT powder could emit strong blue–green fluorescence, whereas no fluorescence emissions appeared for the Na^+^-MMT powder. Moreover, the fluorescence quantum yield was significantly enhanced from 5.79% (TPE-DTAB solution) to 42.10% (TPE-DTAB-modified MMT powder), demonstrating that the intramolecular motions of the AIE-active TPE-DTA^+^ ions were tightly restrained by the rigid framework of MMT layers to suppress the non-radiative decay^56^. Therefore, the as-prepared TPE-DTAB-modified MMT powder was endowed not only a hydrophobic property but also an excellent fluorescence-enhanced performance, distinguishing from conventional fluorophores (aggregation-caused quenching effect).

### Macrodispersion of MMT in PVC/MMT composite

**Figure 4:**
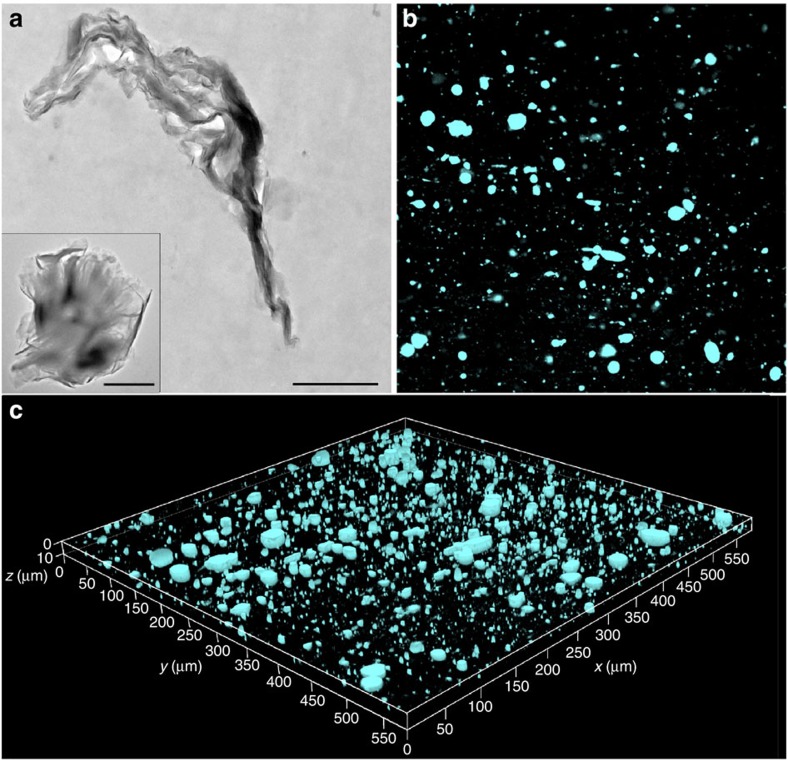
Macrodispersion of organo-modified MMT in PVC/MMT composite. (**a**) Cross-sectional TEM micrograph of PVC/TPE-DTAB-modified MMT (5 wt%) composite; the inset showed TEM image of TPE-DTAB-modified MMT. Scale bar, 1 μm. (**b**) Fluorescence microscopy image (600 × 600 μm^2^) of PVC/TPE-DTAB-modified MMT (5 wt%) composite. (**c**) 3D representation of TPE-DTAB-modified MMT dispersion (cyan parts) in PVC matrix. All fluorescence microscopy images were taken with a 405-nm laser.

The PVC/TPE-DTAB-modified MMT (5 wt%) composite was prepared to investigate the macrodispersion of MMT fillers in PVC matrix by TEM and CFM, respectively. TEM image was obtained from the lateral slice of the PVC/TPE-DTAB-modified MMT composite for the morphological characterization and microdispersion of TPE-DTAB-modified MMT particles in PVC matrix (Fig. 4a). However, TEM observation area was not large enough, and the analysis of microdispersion state was usually dependent on the cut cross-section. In contrast, the as-prepared PVC/MMT composite can be directly observed through CFM by means of the strong AIE-active fluorescence emissions of TPE-DTAB-modified MMT particles. As shown in Fig. 4b, the dark background (600 × 600 μm^2^) was dotted with hundreds of luminescent particles to provide a fluorescence distribution map, clearly demonstrating the macrodispersion state (*XY* plane) of the TPE-DTAB-modified MMT particles in PVC. Furthermore, a total of 30 images in the *XY* plane were collected at different depths using Z-scan technique. As a result, the spatial distribution of TPE-DTAB-modified MMT particles in PVC matrix was achieved by combining these obtained *XY*-plane images (Fig. 4c). A large area can be observed in such images, and thus the imaging of the real dispersed state of MMT was captured, facilitating an impartial judgement of the overall dispersion state of the composite material. More importantly, this preparation-free imaging method is very convenient and time saving (∼5 s for one picture), similar to an ultrafast and non-invasive computed tomography scan to macrodispersion of the composite material.

On the other hand, the dispersion of TPE-DTAB-modified MMT was compared with that of traditional organoclays. Herein, we incorporated TPE-DTAB as a small percentage of guests in traditional organo-MMT (that is, cetyltrimethyl ammonium bromide (CTAB)-modified MMT). Then, the PVC/CTAB/TPE-DTAB-modified MMT (5 wt%) composite was prepared for dispersion evaluation by CFM. As shown in Supplementary Fig. 10, with the help of the fluorescent TPE-DTAB, we could also directly observe the spatial distribution of CTAB/TPE-DTAB-modified MMT by CFM. In comparison with PVC/TPE-DTAB-modified MMT composite, PVC/CTAB/TPE-DTAB-modified MMT composite exhibited a similar dispersion state, although its fluorescence intensity was much lower as a result of small amounts of TPE-DTAB.

### Macrodispersion of LDH in PVC/LDH composite

In addition, another popular organic–inorganic composite, PVC/LDH composite^57^,^58^, was also investigated to verify the generality of the developed visualization strategy. Similarly, the positively charged LDHs were organically modified by an AIE-active anionic surfactant (TPE-SDS) to possess both organic compatibility and AIE characteristic. Supplementary Fig. 11 showed the TEM image of the LDH microdispersion in PVC matrix. On the other hand, the spatial distribution of LDHs were also directly visualized by CFM (Supplementary Fig. 12). These results demonstrated that the proposed visualization strategy exhibited the generality for studying the spatial distribution of inorganic fillers in organic matrix.

## Discussion

In conclusion, we have synthesized a new fluorescent surfactant with AIE effect through attachment of TPE units onto cationic surfactants. The resulting cationic surfactant TPE-DTAB facilitates the construction of organo-modified MMT in order to be compatible with polymer matrix. Furthermore, the synthesis of TPE-DTAB surfactant enables us to develop a simple and versatile fluorescence imaging platform for direct observation of macrodispersion of MMT fillers in polymer matrix. The generality of this strategy further highlights 3D visualization of LDH filler macrodispersion in PVC matrix. Note that scanning electron microscopy could give microscale imaging with simple sample preparation. However, scanning electron microscopy is usually used for investigating surface topography. With the help of penetration depth of electrons, it might also observe dispersion of particles in slight interior of samples. In comparison to the nanoscale electron microscopy techniques (for example, TEM and 3D-TEM tomography), the AIE-active CFM technology offers the advantages of a wide-view dispersion image with simplicity, sensitivity and high-contrast, but a limited spatial resolution of fine structure by light diffraction (∼200 nm in the lateral dimension and 500 nm in the axial dimension)^15^,^17^. In contrast, super-resolution microscopy imaging techniques, such as stimulated emission depletion microscopy and stochastic optical reconstruction microscopy, can obtain a higher resolution than the diffraction limit. For example, stimulated emission depletion microscopy can achieve an image resolution of 20–40 nm in the lateral dimensions and 30–50 nm in the axial dimension^17^; while stochastic optical reconstruction microscopy can reach 20–30 nm and 50–60 nm for the lateral resolution and axial resolution, respectively^59^. It is worth mentioning that super-resolution microscopy is potentially applicable to the proposed AIE method if we can synthesize some AIE molecules suitable for such technique. On the other hand, cathodoluminescence by the interaction between electron beams and solid luminescent materials is a similar idea for the proposed AIE method, which has been widely used to investigate the nanoscale properties of solid samples^60^. Specially, the defect structure of solid samples could be visualized by cathodoluminescence microscopy^61^, which is difficult for the proposed AIE method. In addition, the proposed AIE molecules in our study are designed for charged inorganic and organic substances, which is inapplicable to non-charged inorganic and organic substances. However, it has been reported that non-charged inorganics could be easily modified by luminescent polymers through physisorption^62^. With these merits in the current protocol, this facile imaging platform may open viable opportunities and inspirations for macrodispersion of other inorganic fillers in organic–inorganic composites, especially for dispersion of composite materials difficult to be distinguished using electron microscope imaging.

## Methods

### Materials

Zinc dust and 4-hydroxyl benzophenone were purchased from Sigma-Aldrich Chemical Co. Sodium hydride (NaH), TiCl_4_, poly(vinyl chloride) with Mn=67,750 (polydispersity index (PDI)=2.32), dioctyl phthalate (DOP), CTAB, calcium stearate, zinc stearate, NaCl and K_2_CO_3_ were purchased from J&K Chemical Ltd. Anhydrous dimethyl formamide (DMF), tetrahydrofuran (THF) and sodium sulfate were purchased from Alfa Aesar. 1-Bromooctane, 1,4-dibromobutane and trimethylamine in THF (100 ml, 1.0 M) were purchased from Tokyo Chemical Industry. Ethanol, PE, acetone, ethyl acetate (EA) and dichloromethane were purchased from Beijing Chemical Reagent Company. Sodium montmorillonite (Na^+^-MMT) with cation exchange capacity values of 145 meq per 100 g (from Nanocor, PGW grades) was used without further purification. Carbonate intercalated Mg-Al LDHs (with a molar ratio of 2:1 between Mg^2+^ and Al^3+^) were synthesized and characterized according to the literature^54^. TPE-SDS was synthesized and characterized according to our previous work^36^. All reagents were of analytical grade and used without further purification. Water was purified with a Milli-Q purification system (Milli-Q).

### Synthesis of compound **1**

In a 250-ml, two-necked, round-bottom flask equipped with a condenser, zinc dust (2.9 g, 44 mmol) and 4-hydroxybenzophenone (2.0 g, 10 mmol) were dissolved in 100 ml dry THF under nitrogen. The mixture was cooled to −78 °C and TiCl_4_ (2.5 ml, 22 mmol) was added dropwise. After the addition, the mixture was allowed to warm to room temperature in 0.5 h, and then was heated to reflux for overnight. The reaction was quenched with 10% aqueous K_2_CO_3_ solution. The mixture was extracted with diethyl ether for three times and the combined organic layer was washed with brine twice and dried over sodium sulfate. After solvent evaporation, the crude product was separated through silica-gel chromatography flushed with PE/EA (v/v 1:1). 1.56 g product was obtained as a white solid with a yield of 86%. ^1^H NMR (600 MHz, CDCl_3_, *δ*): 6.94−7.05 (m, 10H), 6.76−6.81 (m, 4H), 6.47−6.52 (m, 4H).

### Synthesis of compound **2**

Under N_2_ atmosphere, sodium hydride (0.022 g, 0.55 mmol) was added to the solution of **1** (0.182 g, 0.50 mmol) in dry DMF (10 ml) and the mixture was stirred for extra 30 min at room temperature. Then 1-bromooctane (0.145 g, 0.75 mmol) was added and the reaction mixture was stirred at 60 °C for 8.0 h. When the reaction completed, the solvent DMF was removed and the residue was redissolved with EA, and the resulting solution was washed with water for three times and dried over sodium sulfate. The solution was concentrated and the residue was purified through silica gel chromatography flushed with PE/EA (7:1 v/v), compound **2** (0.121 g, 0.25 mmol) as a yellow liquid was obtained with a yield of 51%. ^1^H NMR (600 MHz, CDCl_3_, *δ*): 6.98−7.11 (m, 10H), 6.83−6.92 (m, 4H), 6.52−6.63 (m, 4H), 3.82−3.88 (m, 2H), 1.67−1.76 (m, 2H), 1.37−1.41 (m, 2H), 1.24−1.29 (m, 8H), 0.85−0.88 (t, 3H).

### Synthesis of compound **3**

The compound **2** (0.174 g, 0.50 mmol) and K_2_CO_3_ (0.076 g, 0.55 mmol) were mixed in 20 ml acetone. After stirred for 1 h, 1,4-dibromobutane (0.118 g, 0.55 mmol) was added and the resulting mixture was stirred for 24 h at 60 °C. After evaporation of acetone, the obtained solids were first redispersed in EA and then filtered to remove the insoluble K_2_CO_3_. The purified product was obtained in 74% yield after purification separation by silica gel chromatography using PE/EA (10:1 (v/v)). ^1^H NMR (600 MHz, CDCl_3_, *δ*): 7.00−7.12 (m, 10H), 6.88−6.95 (m, 4H), 6.59−6.65 (m, 4H), 4.20−4.25 (m, 2H), 3.84−3.93 (m, 4H), 2.43−2.46 (m, 2H), 1.81−1.86 (m, 4H), 1.70−1.76 (m, 2H), 1.38−1.43 (m, 2H), 1.26−1.32 (m, 8H), 0.87−0.89 (t, 3H).

### Synthesis of TPE-DTAB (compound **4**)

A 100-ml flask with a magnetic spin bar was charged with **3** (0.3 g, 0.50 mmol) dissolved in 20 ml of THF. To this solution, trimethylamine (1.0 M, 5 ml) was added. The mixture was heated to reflux and stirred for 3 days. During this period, 5 ml of trimethylamine in THF was added at several intervals. After THF and extra trimethylamine were evaporated, the residue was washed with chloroform and acetone and then dried overnight *in vacuo* at 60 °C. A yellowish product was obtained in 88% yield. ^1^H NMR (600 MHz, DMSO-d_6_, *δ*): 7.07−7.15 (m, 6H), 6.92−6.98 (m, 4H), 6.80−6.88 (m, 4H), 6.64−6.73 (m, 4H), 3.91−3.95 (m, 2H), 3.82−3.87 (m, 2H), 3.34−3.39 (m, 2H), 3.07−3.09 (t, 9H), 1.79−1.85 (m, 2H), 1.61−1.72 (m, 4H), 1.34−1.36 (m, 2H), 1.24−1.27 (m, 8H), 0.84−0.86 (t, 3H). ^13^C NMR (600 MHz, DMSO-d_6_, *δ*): 157.54, 157.29, 144.27, 144.20, 139.82, 139.64, 136.22, 132.41, 132.36, 131.19, 128.28, 128.17, 126.79, 126.76, 114.23, 114.19, 114.14, 114.09, 67.67, 66.97, 65.40, 52.60, 31.69, 29.20, 29.16, 29.12, 26.11, 26.02, 25.99, 22.55, 19.72, 14.43. MS: *m*/*z*: 590.3996 ([M-Br]^+^, calculated for C_41_H_52_NO_2_, 590.3993).

### Synthesis of TPE-DTAB-modified MMT and TPE-SDS-modified LDH

TPE-DTAB-modified MMT was prepared from Na^+^-MMT by ion exchange method. Typically, a 0.5 g portion of Na^+^-MMT was mixed with 50 ml of deionized water. Then, 0.5 g of TPE-DTAB was added in the MMT solution. The ion exchange was carried out under stirring for 1 h at 60 °C. Then, the reaction solution was centrifuged at 5,000 r.p.m. for 5 min, and the precipitate was washed with distilled water to remove the physically adsorbed TPE-DTAB. The obtained TPE-DTAB-modified MMT was dried under vacuum at 60 °C, and then finely powdered in an agate mortar for further use. TPE-SDS-modified LDH powder was prepared according to the same procedure.

### Synthesis of CTAB/TPE-DTAB-modified MMT

Na^+^-MMT (0.5 g) was dispersed in 50 ml of deionized water. Then, a mixture of TPE-DTAB (0.05 g) and CTAB (0.25 g) was added to the MMT dispersion and stirred for 1 h at 60 °C. After centrifugation and washing, the obtained CTAB/TPE-DTAB-modified MMT solid was dried under vacuum at 60 °C and ground into powder.

### Preparation of PVC/MMT composite

The PVC/TPE-DTAB-modified MMT composite, containing 10.0 g PVC powder, 5.0 g DOP and 0.5 g TPE-DTAB-modified MMT powder, was prepared by blending in a heated double-roller mixer for 5 min at 140 °C. The resulting composites were molded at 120 °C and then cooled at room temperature to give thin films with a thickness of 1 mm. The PVC/CTAB/TPE-DTAB-modified MMT composite was prepared according to the same procedure.

### Preparation of PVC/LDH composite

The PVC/TPE-SDS-modified LDH composite, containing 10.0 g PVC powder, 5.0 g DOP, 0.2 g TPE-SDS-modified LDH powder, 0.23 g calcium stearate and 0.1 g zinc stearate, was prepared by blending in a heated double-roller mixer for 5 min at 140 °C. The resulting composite was molded at 120 °C and then cooled at room temperature to give thin films with a thickness of 1 mm.

### TEM sample preparation

The composite films were ultrathin-sectioned with a diamond knife at −120 °C using a Leica EM UC6 ultramicrotome. The obtained ultrathin sections were then collected in a trough filled with deionized water and placed on 200-mesh copper grids.

### Characterization

Proton and carbon-13 nuclear magnetic resonance (^1^H NMR and ^13^C NMR) spectra were recorded at room temperature with a 600-MHz Bruker spectrometer (Bruker). MS was carried out with Quattro microtriple quadrupole mass spectrometer (Waters). Electrical conductivity measurements were performed using a EC 215 conductivity meter (Shanghai Jingmi Instrumental Co.). TEM photographs were performed on a Tecnai G220 TEM (FEI Company) at an accelerating voltage of 200 kV. Ultraviolet–visible spectra were measured on a USB 4000 miniature fibre optic spectrometer in absorbance mode with a DH–2000 deuterium and tungsten halogen light source (Ocean Optics). Fluorescence spectra were obtained using a F–7000 fluorescence spectrophotometer at a slit of 5.0 nm with a scanning rate of 1,200 nm min^−1^. X-ray diffraction measurements of MMT and TPE-DTAB-modified MMT were performed with a Brucker D8 ADVANCE X-ray diffractometer (Bruker) equipped with graphite–monochromatized Cu/Kα radiation (*λ*=0.1541, nm). The samples as unoriented powders were step-scanned in steps of 0.02° (2*θ*) in the range of 2–10°. Fourier transform infrared spectroscopy experiments were carried out on Nicolet 380 system (Thermo) containing a controlled environment chamber equipped with CaF_2_ windows. Zeta potential was determined using a Malvern Zetasizer 3000HS nano-granularity analyzer. The quantum yield values were obtained from the reconvolution fit analysis (Edinburgh F980 analysis software) equipped with an integrating sphere. Fluorescence microscope images were recorded on a confocal laser scanning microscope (Leica, TCS SP8).

### Data availability

The data that support the findings of this study are available from the corresponding author upon request.

## Additional information

**How to cite this article:** Guan, W. *et al*. Fluorescence microscopy as an alternative to electron microscopy for microscale dispersion evaluation of organic–inorganic composites. *Nat. Commun.* 7:11811 doi: 10.1038/ncomms11811 (2016).

## Supplementary Material

Supplementary Information Supplementary Figures 1-12

## Figures and Tables

**Figure 1:**
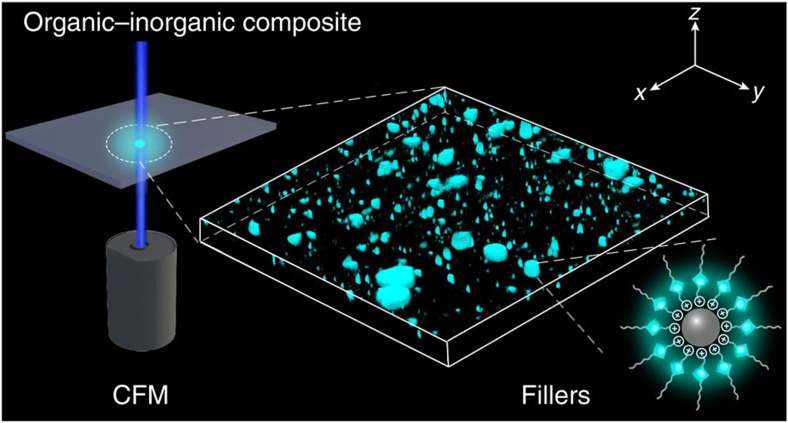
Schematic representation of visualization of 3D macrodispersion of fillers in organic–inorganic composites. The inorganic fillers modified and bound with AIE molecules are dispersed inside the organic matrix, and then directly visualized by CFM.
