# Supplementary material for: Fluorescence microscopy as an alternative to electron microscopy for microscale dispersion evaluation of organic–inorganic composites
Source: Nat Commun. 2016 Jun 2;7:11811. doi: 10.1038/ncomms11811 (PMC4895723; doi:10.1038/ncomms11811)
Supplement: Supplementary Information — Supplementary Figures 1-12 [file ncomms11811-s1.pdf]

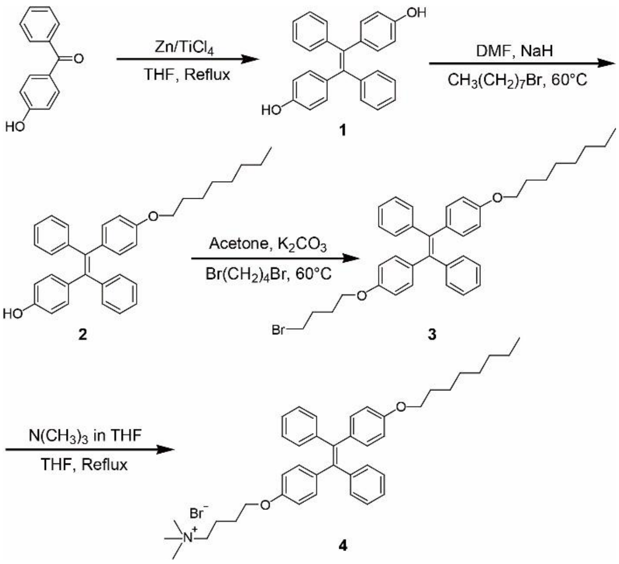

Supplementary Figure 1 | Synthetic route of amphipathic TPE-DTAB (4). THF = tetrahydrofuran, DMF = N, N-dimethylformamide.

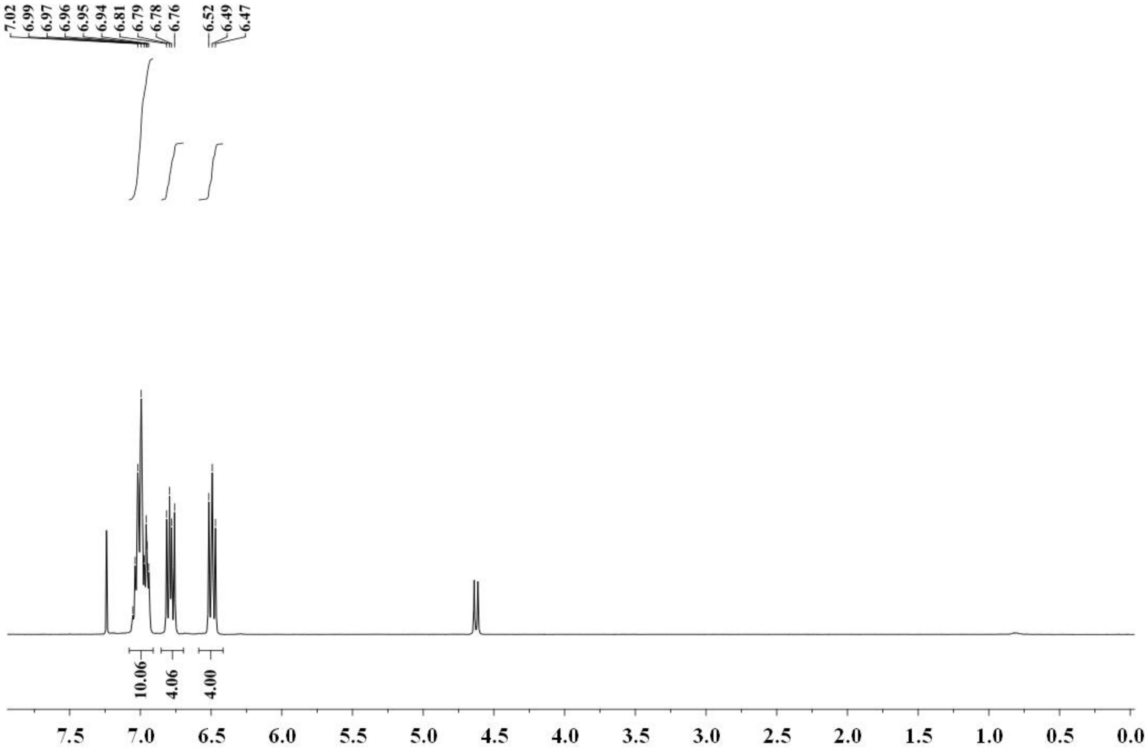

Supplementary Figure 2 | ^{1}H NMR spectrum of 1 in $CDCl_3$.

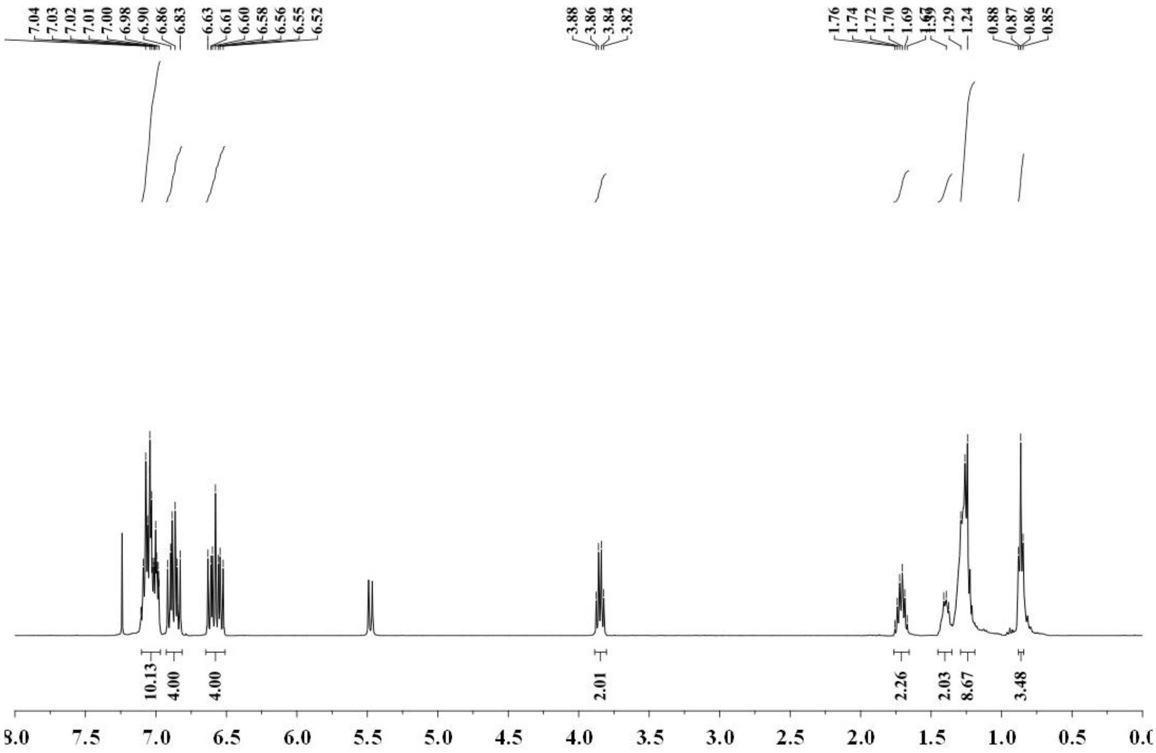

Supplementary Figure 3 | ^{1}H NMR spectrum of 2 in $CDCl_3$.

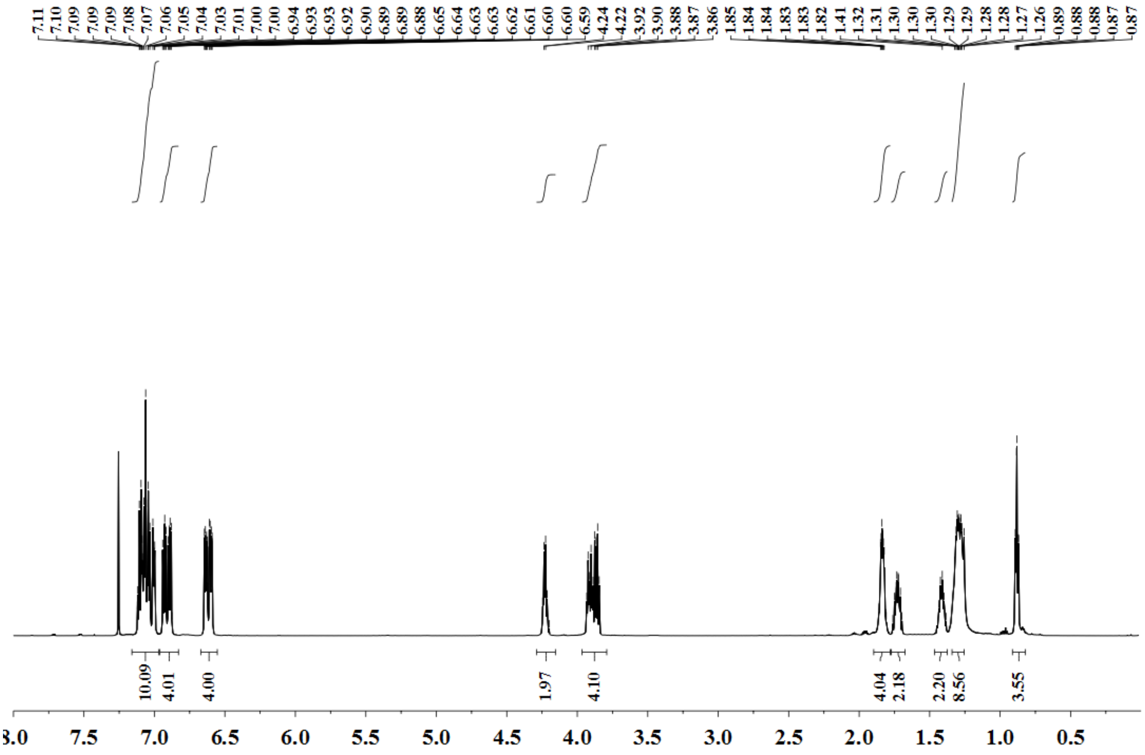

Supplementary Figure 4 | ^{1}H NMR spectrum of 3 in $CDCl_3$.

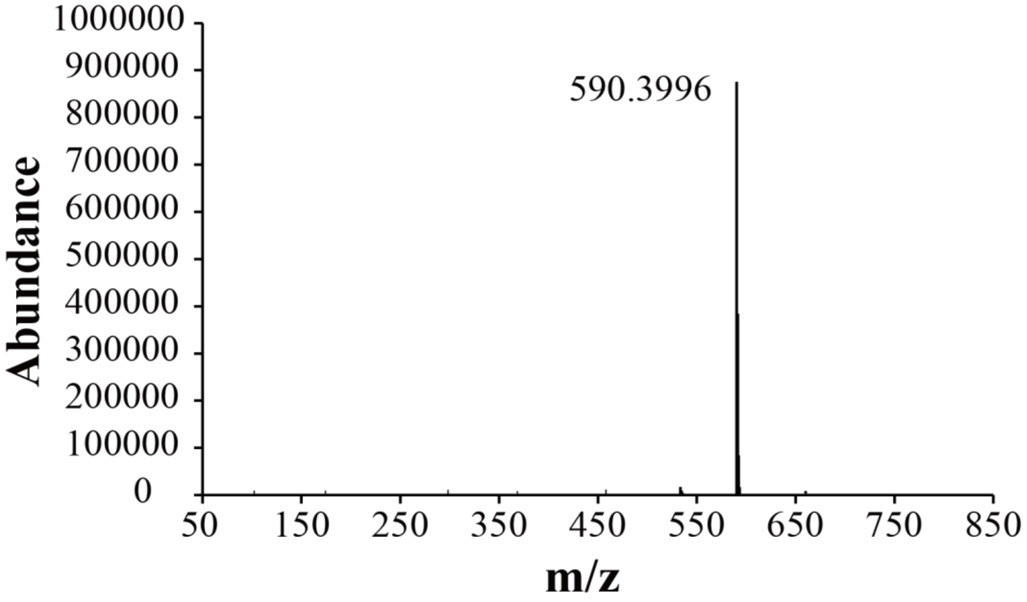

Supplementary Figure 5 | Positive-ion mode ESI-MS spectrum of TPE-DTAB (4).

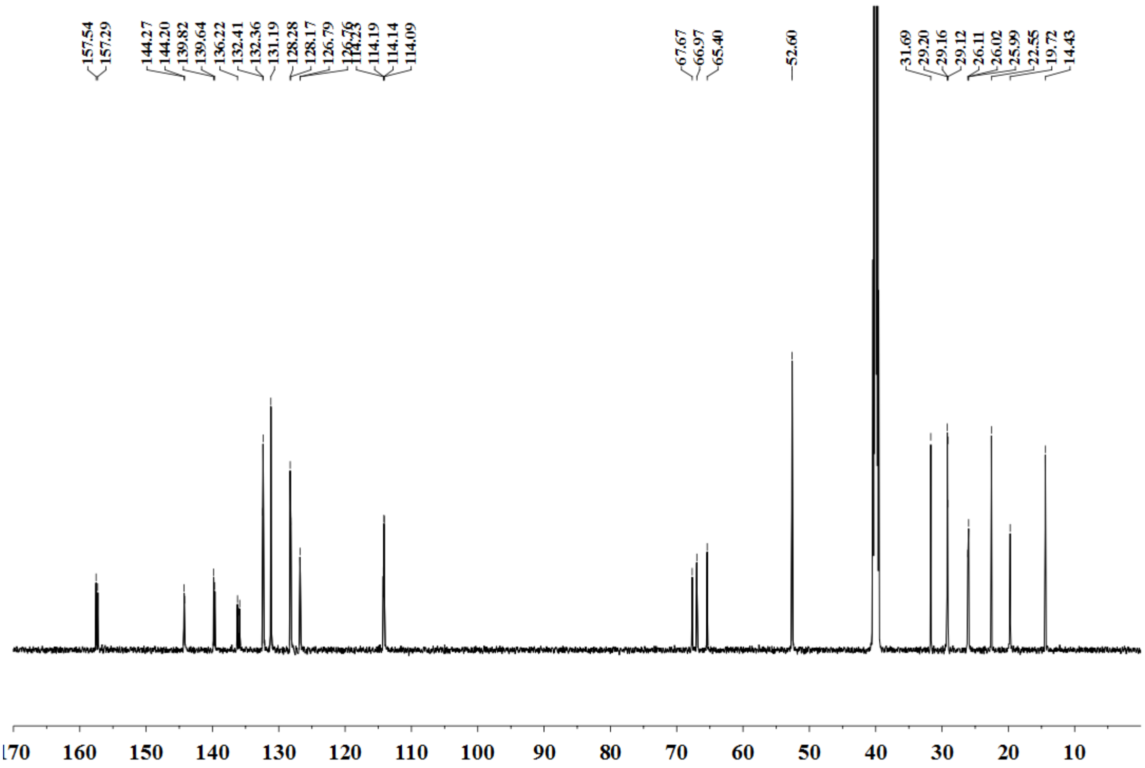

Supplementary Figure 6 | ^{13}C NMR spectrum of TPE-DTAB (4) in DMSO-d_6.

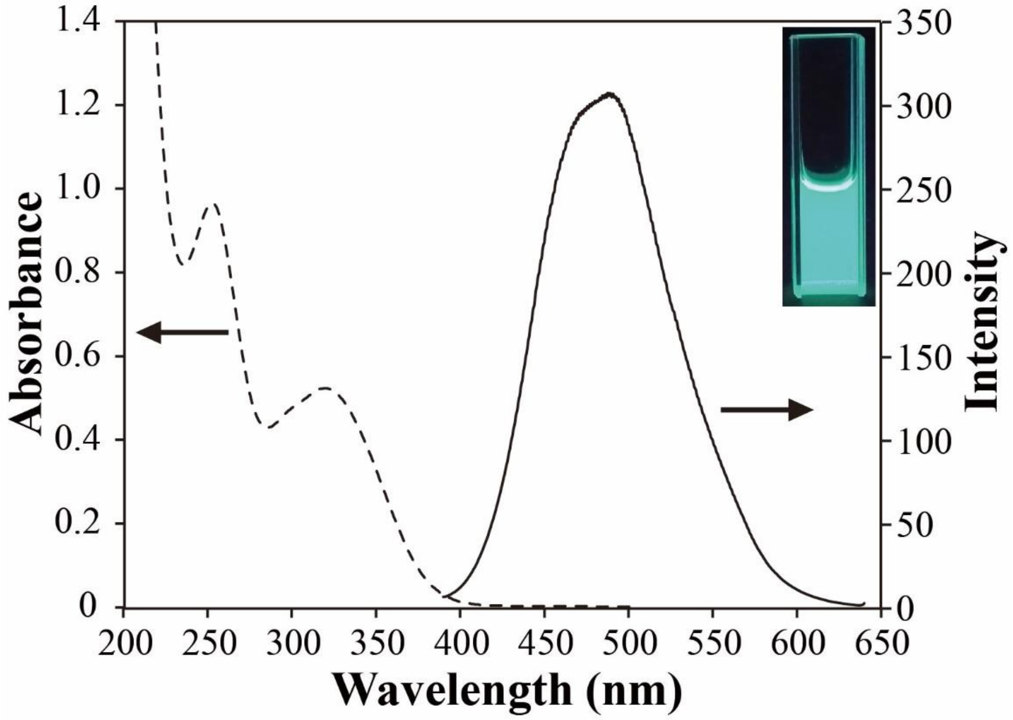

Supplementary Figure 7 | Optical properties of TPE-DTAB. UV−visible absorption (dotted line) and fluorescence (solid line) spectra of 40 μM TPE-DTAB aqueous solution.

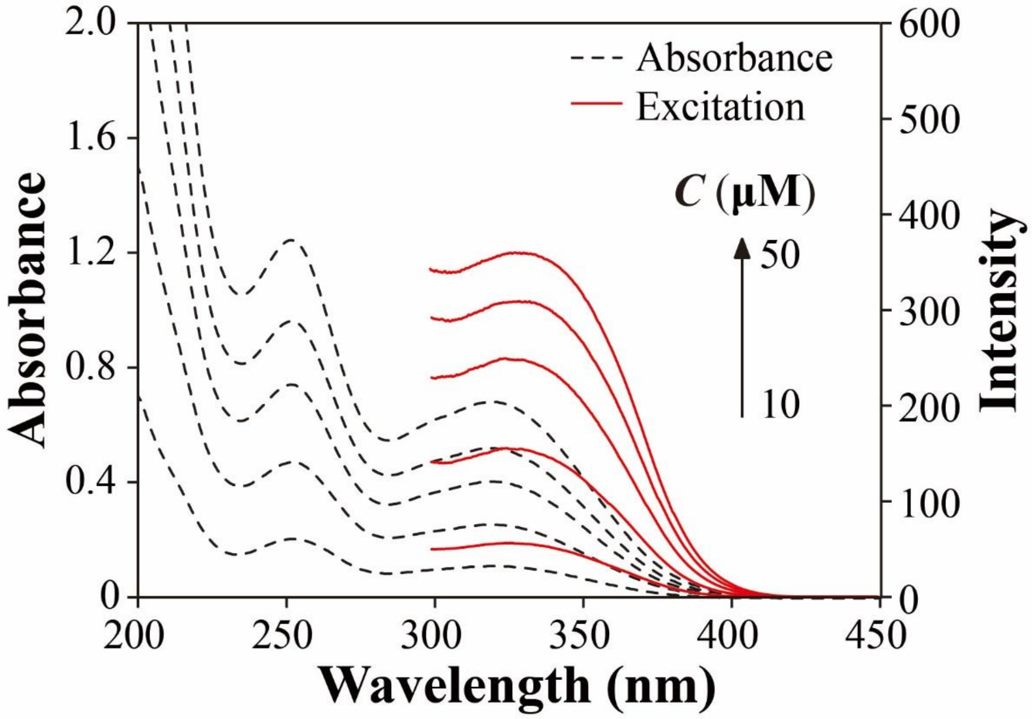

Supplementary Figure 8 | Optical properties of TPE-DTAB below and above CMC. UV absorption (dotted line) and fluorescence excitation (solid line) spectra of TPE-DTAB at 10, 20, 30, 40, and 50 μM, respectively.

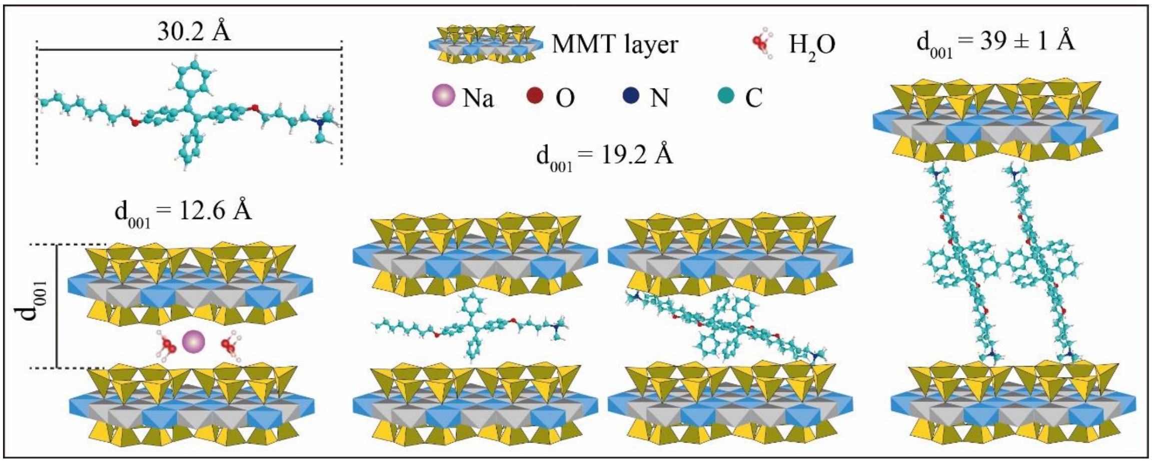

Supplementary Figure 9 | Schematic representation of the possible arrangement of TPE-DTAB molecules within the MMT layer space.

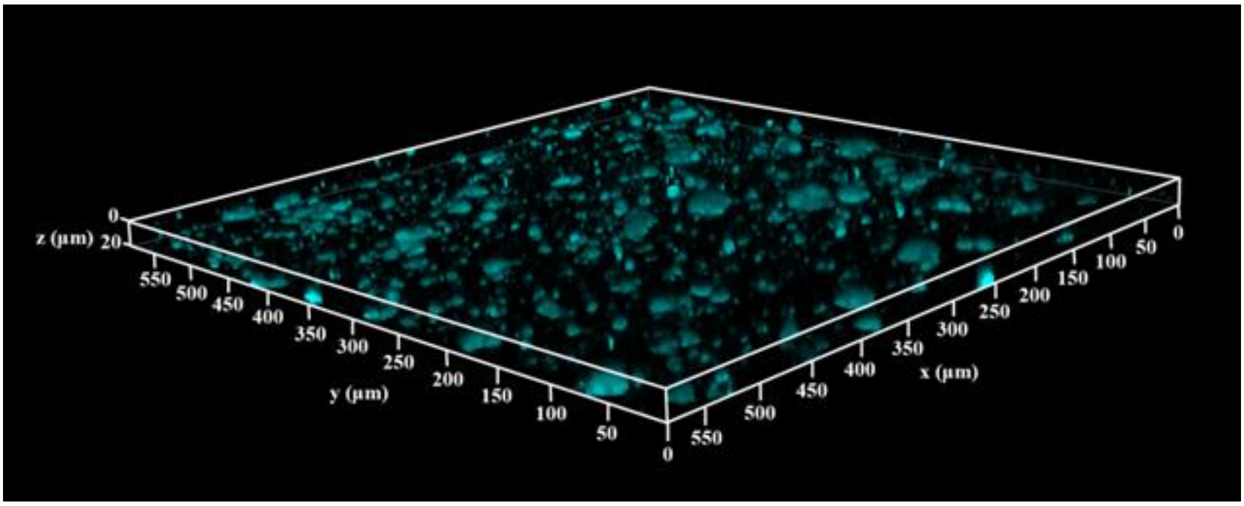

Supplementary Figure 10 | CFM image of PVC/CTAB/TPE-DTAB modified MMT composite. The image shows 3D-representation of CTAB/TPE-DTAB modified MMT dispersion (cyan parts) in the PVC/CTAB/TPE-DTAB modified MMT (5 wt%) composite.

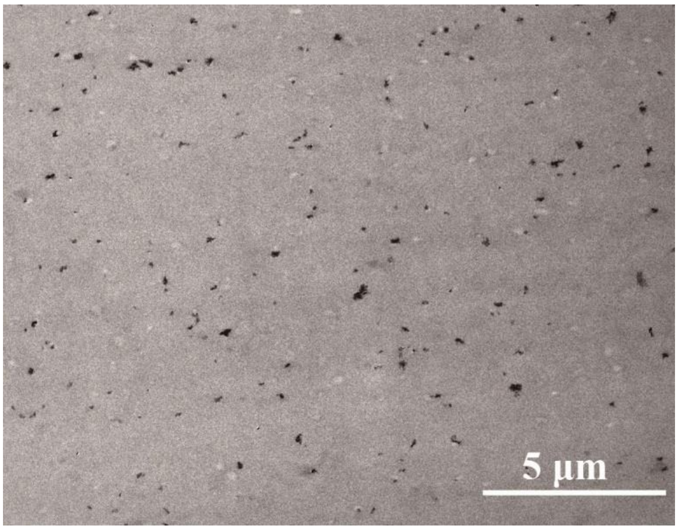

Supplementary Figure 11 | Cross-sectional TEM micrograph of PVC/TPE-SDS modified LDH (2 wt%) composite.

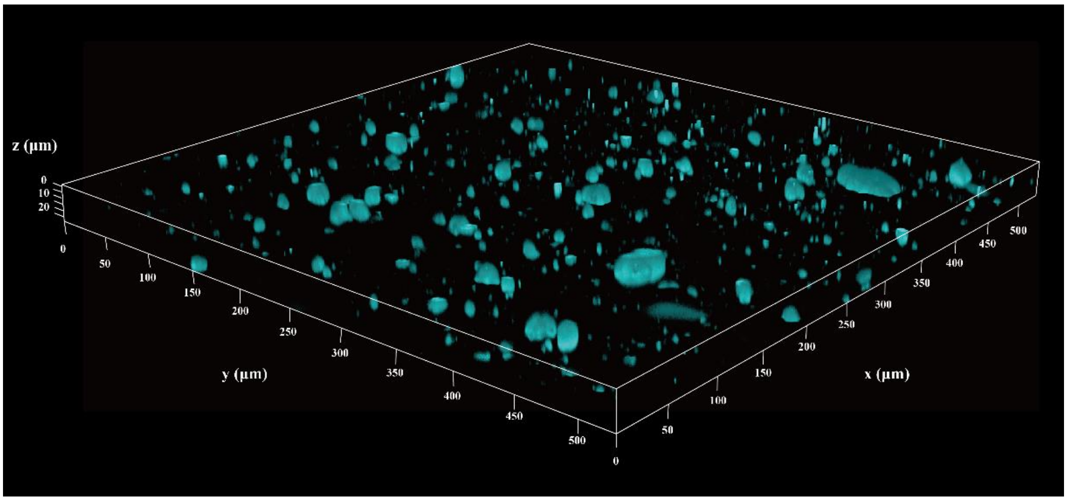

Supplementary Figure 12 | CFM image of PVC/TPE-SDS modified LDH composite. The image shows 3D-representation of TPE-SDS modified LDH dispersion (cyan parts) in the PVC/TPE-SDS modified LDH (2 wt%) composite.
